# The Role of Micro CT in the Imaging of Cancer

**DOI:** 10.1155/2014/608206

**Published:** 2014-12-25

**Authors:** M. Griffin, P. Anand, R. Tang, A. Zambeli-Ljepovic, M. Senter-Zapata, M. Lewin-Berlin, A. Bui, J. Singh, K. Patel, W. Sarraj, J. Gilbertson, Y. Yagi, D. Kopans, R. Moore, A. Ly, M. Saksena, G. P. Nielsen, G. Harris, N. Gershenfeld, B. Smith, J. Michaelson

**Affiliations:** Departments of Surgery and Pathology, Massachusetts General Hospital, 65 Landsdowne Street, 2nd Floor, Cambridge, MA 02139, USA

## Introduction

The absence of real-time, detailed, 3D information on the composition of surgical specimens presents an enormous challenge in surgical oncology and pathology.

## Background

The problem is especially pressing for breast cancer, where as many as ~1 in 3 patients undergoing lumpectomy have been found, upon pathological examination of the slides, to be margin positive. These patients need to return to the hospital for reexcision, sometimes multiple times, in order to achieve negative margins.

## Aims

A solution may be found in a relatively new technology, Micro CT, a high resolution X-ray imaging method, that has been widely used in industry and materials science but little used in medicine.

## Materials and Methods

Over the past three years, we have imaged a great variety of surgical specimens with three Micro CT machines (a SkyScan 1173 Micro CT, an Xradia MicroXCT-200, and a Nikon Metrology XTH225).

## Results

Our findings indicate that the Micro CT is able to provide 3D images of surgical specimens, which can identify, within 10 minutes, most of those breast cancer patients later found to be margin positive on pathological analysis as well as to identify a small number of patients whose cancers appear to be margin positive on Micro CT alone. Micro CT can also identify lymph nodes in cancer specimens, including nodes not detected by pathological dissection.

## Conclusion

These findings suggest that Micro CT has a considerable potential for providing the surgeon and pathologist with rapid, accurate, actionable information on the status of the surgical specimen while the patient is still in the OR.

